# Novel mechanistic insights of the potential role of gasotransmitters and autophagy in the protective effect of metformin against hepatic ischemia/reperfusion injury in rats

**DOI:** 10.1007/s00210-025-03837-1

**Published:** 2025-02-06

**Authors:** Ahmed O. Abdel-Zaher, Marwa H. Bakr, Yomna H. Gad, Alaa T. Abdelhafez

**Affiliations:** 1 Department of Pharmacology, Faculty of Medicine, Assiut University, Assiut, Egypt; 2 Department of Histology and Cell Biology, Faculty of Medicine, Assiut University, Assiut, Egypt; 3 Department of Basic Medical Science, Badr University, Assiut, Egypt; 4 Department of Pharmacology and Toxicology, Faculty of Pharmacy, Assiut University, Assiut, Egypt

**Keywords:** Metformin, Hepatic ischemia/reperfusion injury (I/RI), CO, H_2_S, NO, Autophagy

## Abstract

Metformin exerts antidiabetic and pleiotropic effects. This study investigated the function and mechanisms of gasotransmitters and autophagy in the metformin-induced protection against ischemia/reperfusion injury (I/RI). According to measurements of serum hepatic function indicators and histopathological evaluation, metformin protected against hepatic I/RI-induced impairment of liver function and structure. In addition, metformin inhibited hepatic I/RI-induced hepatic oxidative stress, nitrosative stress, inflammation, and apoptosis. Also, it suppressed hepatic I/RI-induced decrease in hepatic heme oxygenase-1 (HO-1) and hydrogen sulfide (H_2_S) levels and increase in nitric oxide (NO) production. Furthermore, metformin inhibited hepatic I/RI-induced decrease in protein expressions of endothelial NO synthase (eNOS), HO-1, cystathionine γ-lyase (CSE), and Beclin-1 and increase in the protein expression of inducible NO synthase (iNOS) in the liver tissue. Co-administration of the NO biosynthesis inhibitor, L-NAME, carbon monoxide(CO)-releasing molecule-A_1_ (CORM-A_1_), the H_2_S donor, NaHS, or the autophagy stimulator, rapamycin (RAPA), enhanced all effects of metformin. The NO donor, L-arginine, the CO biosynthesis inhibitor, zinc protoporphyrin, the H_2_S biosynthesis inhibitor, DL-propargylglycine, or the autophagy inhibitor, chloroquine (CQ), antagonized the effects of metformin. These findings reveal, for the first time, that increasing CO, H_2_S, and autophagy levels with subsequent decreasing NO level play a critical role in metformin's protective action against hepatic I/RI. The ability of L-NAME, CORM-A_1_, NaHS, and RAPA to boost metformin’s protective effect in hepatic I/RI may positively be attributed to their ability to lower hepatic oxidative stress, nitrosative stress, inflammation, and apoptosis.

## Introduction

Hepatic ischemia reperfusion injury (I/RI) is a serious complication caused and implicated in the pathogenesis of several clinical situations, such as liver transplantation or resection, hemorrhagic shock, and severe trauma (Mao et al. 2021)*.* The hepatic I/RI involves a complicated series of destructive processes including oxidative stress, nitrosative stress, apoptosis, and inflammation which are initiated in ischemic phase and subsequently aggravated in reperfusion phase (Neri et al. 2020).

During the I/R process, oxidative stress is considered to play a crucial role in I/R damage (Bardallo et al. 2022; Xia et al. 2024). Inhibition of oxidative stress can protect against hepatic I/RI (Jiang et al. 2023; Li et al. 2023). Also, inflammatory cytokines such as tumor necrosis factor-α (TNF-α) and IL-6 are key factors in hepatic I/RI. Inhibition of inflammation alleviates hepatic I/RI (Li et al. 2023; Nakatake et al. 2024). In addition, apoptosis is main type of cell death during hepatic I/RI (Li et al. 2015). Antiapoptotic measures, including the overexpression of antiapoptotic (Bcl-2), down expression of pro-apoptotic (Bax), and caspase-3 inhibition, can protect the liver from I/RI (Yang et al. 2021; Jiang et al. 2024).

Autophagy is a self-digesting process that controls the delivery of an extensive range of malformed proteins and unnecessary cytoplasmic contents into the lysosome for catabolic breakdown (Wang et al. 2019). Enhancing of autophagy may represent an innovative therapeutic strategy to ameliorate hepatic I/RI (Zhang et al. 2022a).

The gaseous signaling molecules, nitric oxide (NO), carbon monoxide (CO), and hydrogen sulfide (H_2_S), play vital roles in both physiological and pathological processes in mammals (Pałasz et al. 2021). Although these gases were considered to be toxic to the environment, it was discovered that these gases are endogenously generated and enzymatically regulated in mammals under normal physiological conditions to exhibit cytoprotective properties (Moody and Calvert 2011).

Nitric oxide is produced endogenously from L-arginine (L-A) by a family of enzymes, called NO synthases (NOS), including neuronal NOS (nNOS or NOS1), inducible NOS (iNOS or NOS2), and endothelial NOS (eNOS or NOS3) (Salihi et al. 2022). Usually, nNOS and eNOS are constitutively expressed and produce low level of NO (McNamara et al. 2014). Conversely, iNOS is mainly induced in pathological conditions leading to the production of NO at much greater levels (Cinelli et al. 2020). It has been reported that eNOS-derived NO is hepatoprotective in I/RI while excessive NO produced by iNOS aggravates hepatic I/RI (Zhang et al. 2022b; Chaabani et al. 2023). Furthermore, Nakatake et al. (2022) found that iNOS was upregulated in hepatic I/RI.

In prior studies using rat experimental models, it was revealed that recipients’ inhalation of CO gas prevents I/RI of the transplanted heart, intestine, liver, and kidney, an effect that was due to the antioxidant, anti-inflammatory and antiapoptotic properties of CO (Abe et al. 2017). However, the therapeutic benefits of CO gas administration have been questioned due to its potential systemic toxicity. The potent cytoprotective and anti-inflammatory effects of CO can be achieved by applying of so-called CO-releasing molecules (CORMs). CORMs as CORM-3 and CORM-A_1_ are known to have a variety of pharmacological and therapeutic activities through liberation of controlled amounts of CO in organs (Iqbal et al. 2021; Obara et al. 2022).

In higher organisms, the stress inducible enzyme, heme oxygenase-1 (HO-1), represents the main source of endogenous CO (Li et al. 2019). A great number of studies have indicated that HO-1/CO system has potent antioxidative, antiapoptotic, anti-inflammatory, and cytoprotective activities against I/RI and is a potential target for treating hepatic I/RI (Li et al. 2019).

In mammalian cells, H_2_S is produced from L-cysteine via two heme-containing enzymes: cystathionine β-synthase (CBS) and cystathionine γ-lyase (CSE). CBS is predominantly responsible for the synthesis of H_2_S in the central nervous system, whereas CSE has higher expression in the kidney and liver (Giuffrè and Vicente 2018). Administration of H_2_S donors as sodium hydrosulfide (NaHS) significantly attenuated the severity of liver injury and inhibited inflammation, apoptosis and oxidative stress, which were elevated by hepatic I/RI (Chen et al. 2021)

The antihyperglycemic medication, metformin, exerts its effects via activation of 5-adenosine monophosphate-activated protein kinase (AMPK) which plays a key regulatory role in the balance of cellular energy (Rena et al. 2017).

It has been found that metformin attenuates myocardial I/RI and bile duct ligation-induced liver injury through antioxidant actions (Wang et al. 2017; Mansourian et al. 2018). Also, in a renal I/RI rat model, metformin can achieve anti-inflammatory and antioxidant effects (Dehkordi et al. 2018). Moreover, metformin has been found to protect rats against cerebral I/RI through lowering neuronal apoptosis and microglial inflammation (Liu et al. 2022). In addition, metformin by enhancing autophagy alleviates hepatic steatosis (Afshari et al. 2023). Furthermore, there is evidence suggesting that metformin through activation of AMPK upregulates HO-1 expression (Kim et al. 2015) and induces eNOS (Sahinturk 2023). However, metformin reduces the expression of iNOS in RAW 264.7 macrophage-like cells (Kato et al. 2010). Moreover, Ma et al. (2020) reported that metformin alleviates atherosclerosis by enhancing H_2_S production via regulating cystathionine γ-lyase (CSE) expression.

In light of these findings, the current study is designed to investigate the potential protective effect of metformin against hepatic I/RI in rats and monitoring the role of oxidative stress, nitrosative stress, inflammation, apoptosis, and autophagy in this effect. In addition, the interrelationship between gasotransmitters, autophagy, and metformin’s protective effect will be evaluated.

## Materials and methods

### Animals

Male adult Wistar rats weighing 220–300 g, purchased from animal house of Faculty of Medicine, Assiut University, were used in all experiments. The animals were housed in stainless steel cages under a 12 h light/dark cycle at 25 °C. Rats were allowed water and food (laboratory chow) *ad libitum*. The study was conducted in accordance with the internationally accepted principles for Guide for the Care and Use of Laboratory Animals. The experiments reported here were approved by our institutional ethics committee (17300541)

### Induction of hepatic ischemia/reperfusion injury (I/RI)

The rats were anaesthetized with an intraperitoneal (i.p.) injection of 1.25 g/kg urethane (25% solution in saline). After shaving and sterilizing the abdomen with betadine, a midline incision was made to expose the liver. The ligaments around the liver were dissected to mobilize the left lobe. The bile duct, the portal vein, and left lateral branch of the hepatic artery (portal triad) were clamped with a microvascular bulldog clamp to occlude the blood flow to the left and median liver lobes resulting in 70% (partial) hepatic ischemia (Dogan et al. 2012; Abdelsameea et al. 2017).

During the ischemic period, which lasts for 60 min, the incision was covered with saline wetting gauze, and the body temperature was maintained with a heating lamp at 37°C. After 60 min of partial hepatic ischemia, the clamp was removed to initiate hepatic reperfusion for 2 h and the laparotomy was sutured using 4-0 silk. Animals in the sham-operated group underwent the same procedure, but without vascular occlusion.

### Experimental protocol

Rats were randomly divided into twelve groups; each group consists of six rats. Rats of group I were treated with 200 mg/kg/day metformin (10 % solution in saline) i.p. for six successive days before induction of ischemia and promptly at the onset of reperfusion. Group II and III rats were treated for six successive days before induction of ischemia and promptly at the onset of reperfusion with 200 mg/kg/day metformin i.p. in combination with 100 mg/kg/day L-arginine [the NO donor, (L-A, 4% solution in saline)] and 10 mg/kg/day L-N(G)-nitro arginine methyl ester [the NO biosynthesis inhibitor (L-NAME, 1% solution in saline)] i.p., respectively. Animals of groups IV and V were treated for six successive days before induction of ischemia and promptly at the onset of reperfusion with 200 mg/kg/day metformin i.p. combined with 0.1 mg/kg/day carbon monoxide-releasing molecule-A_1_ (CORM-A_1_, 0.01% solution in saline) and 0.25 mg/kg/day zinc protoporphyrin [the CO biosynthesis inhibitor (ZnPP, 0.05% solution in saline)] i.p., respectively. Rats of groups VI and VII were treated for six successive days before induction of ischemia and promptly at the onset of reperfusion with 200 mg/kg/day metformin i.p. in combination with 3 mg/kg/day sodium hydrosulfide [the H_2_S donor, (NaHS, 0.3% solution in saline)] and 5 mg/kg/day DL-propargylglycine [the H_2_S biosynthesis inhibitor (DL-PAG, 0.5% solution in saline)] i.p., respectively. Group VIII and IX rats were treated for six successive days before induction of ischemia and promptly at the onset of reperfusion with 200 mg/kg/day metformin i.p. in combination with 1 mg/kg/day rapamycin [the autophagy stimulator, (RAPA, 0.1% solution in saline suspended with 1% CMC)] and 10 mg/kg/day chloroquine [the autophagy inhibitor (CQ, 1% solution in saline)] i.p., respectively.

Control, sham-operated, and I/R animals were treated similarly with the pure vehicle.

In accordance with previous studies, doses of metformin (Yuan et al. 2019), gasotransmitter modulators (Abdel-Zaher et al. 2021), rapamycin (Koizumi et al. 2014), and chloroquine (Dhanabalan et al. 2020) were selected.

### Biochemical measurements

At the completion of experiment, the rats were decapitated. Each animal’s blood and liver tissue were collected. Blood samples were centrifuged for ten min using Eppendorf centrifuge. Following centrifugation, serum samples were taken to estimate alanine aminotransferase (ALT) and aspartate aminotransferase (AST). The serum samples can be used promptly or kept at −80°C until the assay.

The isolated liver from each rat was rinsed with ice-cold saline, and a portion of each liver was preserved in 10 % formalin for histopathological and immunohistochemical examination, while the other portion was dissected, cleaned from the fat, and carefully blotted. The liver tissue was fragmented into small pieces, and an appropriate weight was homogenized in either saline or 10% w/v phosphate buffer (pH 7.4). Two portions of the homogenate were separated. The first portion was centrifuged for 10 min at 10,000 rpm, and the supernatant was utilized to estimate malondialdehyde (MDA), nitrite, tumor necrosis factor-alpha (TNF-α), caspase-3, hydrogen sulfide (H_2_S), and heme oxygenase-1 (HO-1) levels directly or stored at −80°C until assay. An equal volume of perchloric acid (1 mol/l) was added to the second homogenate portion, and the mixture was vortexed. The mixture was left to stand at room temperature for 5 min. After centrifugation for ten min, the supernatant was carefully collected and either utilized directly to estimate intracellular reduced glutathione (GSH) level or kept at −80°C until assay.

The serum ALT and AST levels were measured by kinetic method using the commercially available alanine aminotransferase (ALT/GPT) and aspartate aminotransferase (AST/GOT) liquizyme kits (Spectrum Diagnostics, Egyptian Company for Biotechnology, Cairo, Egypt) according to the manufacturer’s instructions.

Lipid peroxidation was measured in the supernatant using the thiobarbituric acid reactive compounds method, which was previously described by Ohkawa et al. (1979).

Using Ellman’s reagent, the intracellular GSH content in the liver was measured using the method of Ellman (1959).

TNF-α, caspase-3, and HO-1 levels in liver tissue homogenate were measured using the commercially available ELISA kits (Elabscience Biotechnology Inc., USA) following the manufacturer’s instructions.

Nitrite concentration in the supernatant was evaluated by using Griess reagent (Griess 1879) as described by Miranda et al. (2001).

The hepatic level of H_2_S in the supernatant was estimated using the method of Wiliński et al. (2017).

### Histopathological examination

The rats from each group were sacrificed at the end of experimental duration. Livers were fixed in 10% buffered formalin, embedded in paraffin, and then sliced serially into thin sections (5 µm) using a microtome, mounted on slides, and subjected to the following techniques: hematoxylin and Eosin staining for histological assessment and Sirius red staining for evaluation of collagen fibers (Bancroft and Gamble 2008).

The number of normal hepatocytes in a specific area was estimated. At least five microscopic fields were evaluated at magnification ×400 using ImageJ version 1.52 k.

The mean area % of collagen fibers of SR-stained sections was determined. The measurements were performed using a ×10 objective lens in five nonadjacent fields from five distinct sections per group.

### Immunohistochemical analysis

Further sections with a thickness of 3 to 5 µm were sliced and mounted on slides coated with poly L-lysine from the previously prepared paraffin blocks. The slides were dried after mounting to remove any water that might have been trapped under the section. To accomplish this, the slides were left in an oven set at 60°C for the entire night.

Endothelial nitric oxide synthase (eNOS), inducible nitric oxide synthase (iNOS), heme oxygenase-1 (HO-1), cystathionine γ-lyase (CSE), and Beclin-1 were examined immunohistochemically by using the standardized commercially available step plus poly-HRP anti mouse/rabbit IgG detection system (with DAB solution).

For negative control staining, certain sections were incubated with PBS rather than the primary antibody. There was no immunoreactivity in these sections.

Histological and IHC staining was evaluated by an Olympus microscope (CX43, Tokyo, Japan) and was photographed by a camera (CX43, Hamburg, Germany).

In the evaluation of immunohistochemical staining, intensity of staining and distribution of the cells in liver specimens were scored. The intensity was scored with a four-point scale as follows: zero for negative, 1 for weak, 2 for moderate, and 3 for strong. The distribution of positive cells was scored with a 4-point index as follows:1 for 0–25%, 2 for 26–50%, 3 for 51–75%, and 4 for ˃75%. Then, the distribution and intensity were multiplied to calculate the immunoreactivity score.

### Chemicals

L-N(G)-nitroarginine methyl ester (L-NAME), urethane, zinc protoporphyrin (ZnPP), rapamycin, chloroquine diphosphate salt, and N,N-dimethyl-p-phenylenediamine sulfate were obtained from Sigma-Aldrich Co. (USA). Metformin was obtained from CID pharmaceutical factory (Assiut, Egypt). Thiobarbituric acid was obtained from MP Biomedicals Inc. (France), and malondialdehyde bis-(dimethylacetal) was bought from Merck (Germany). Other compounds were all analytically graded.

### Statistical analysis of results

Results were expressed as the mean ± standard error of the mean (X ± S.E.M.). The difference between groups was statistically analyzed using the one-way analysis of variance (ANOVA) followed by the Tukey method test as post hoc analysis. All statistics were performed using the GraphPad Prism software (GraphPad; San Diego CA, USA).

## Results

### Effect of hepatic ischemia/reperfusion (I/R) on the rat liver function and hepatic levels of biochemical parameters

**Fig. 1:**
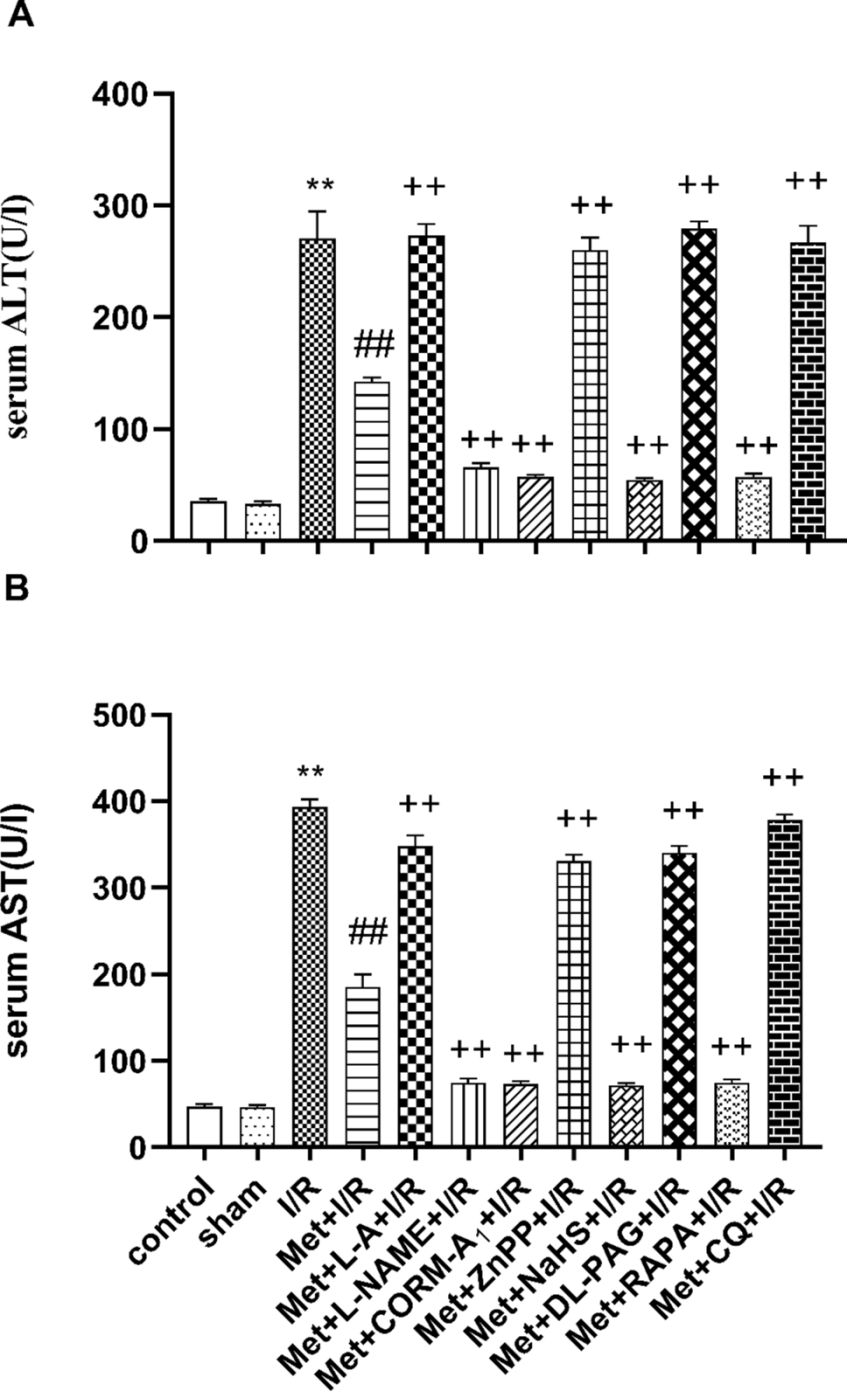
Effect of concomitant administration of nitric oxide (NO), carbon monoxide (CO), hydrogen sulfide (H_2_S), or autophagy modulators with 200 mg/kg/day metformin (Met) i.p. to rats for 6 successive days before induction of ischemia and promptly at the onset of reperfusion on the serum **A** alanine aminotransferase (ALT) and **B** aspartate aminotransferase (AST) levels. Animals were treated with 100 mg/kg/day L-arginine (L-A), 10 mg/kg/day L-N(G)-nitroarginine methyl ester (L-NAME), 0.1 mg/kg/day carbon monoxide releasing molecule-A_1_ (CORM-A_1_), 0.25 mg/kg/day zinc protoporphyrin (ZnPP), 3 mg/kg/day sodium hydrosulfide (NaHS), 5 mg/kg/day DL-propargylglycine (DL-PAG), 1 mg/kg/day rapamycin (RAPA), or 10 mg/kg/day chloroquine (CQ) i.p. concomitantly with Met. To induce hepatic I/R, the rats were subjected to 1 h of ischemia followed by 2 h of reperfusion. Blood samples were collected for biochemical measurements at the end of experimental duration. Each value represents the mean ± S.E.M. of 6 observations. ^**^p< 0.01 vs. control and sham values; ^##^*p*<0.01 vs. I/R values; ^++^*p*<0.01 vs. Met+I/R values

As shown in Fig. 1, hepatic ischemia for 1 h followed by reperfusion for 2 h significantly increased the serum ALT level (Fig. 1A) and AST level (Fig. 1B) as compared to control and sham-operated animals.

**Fig. 2:**
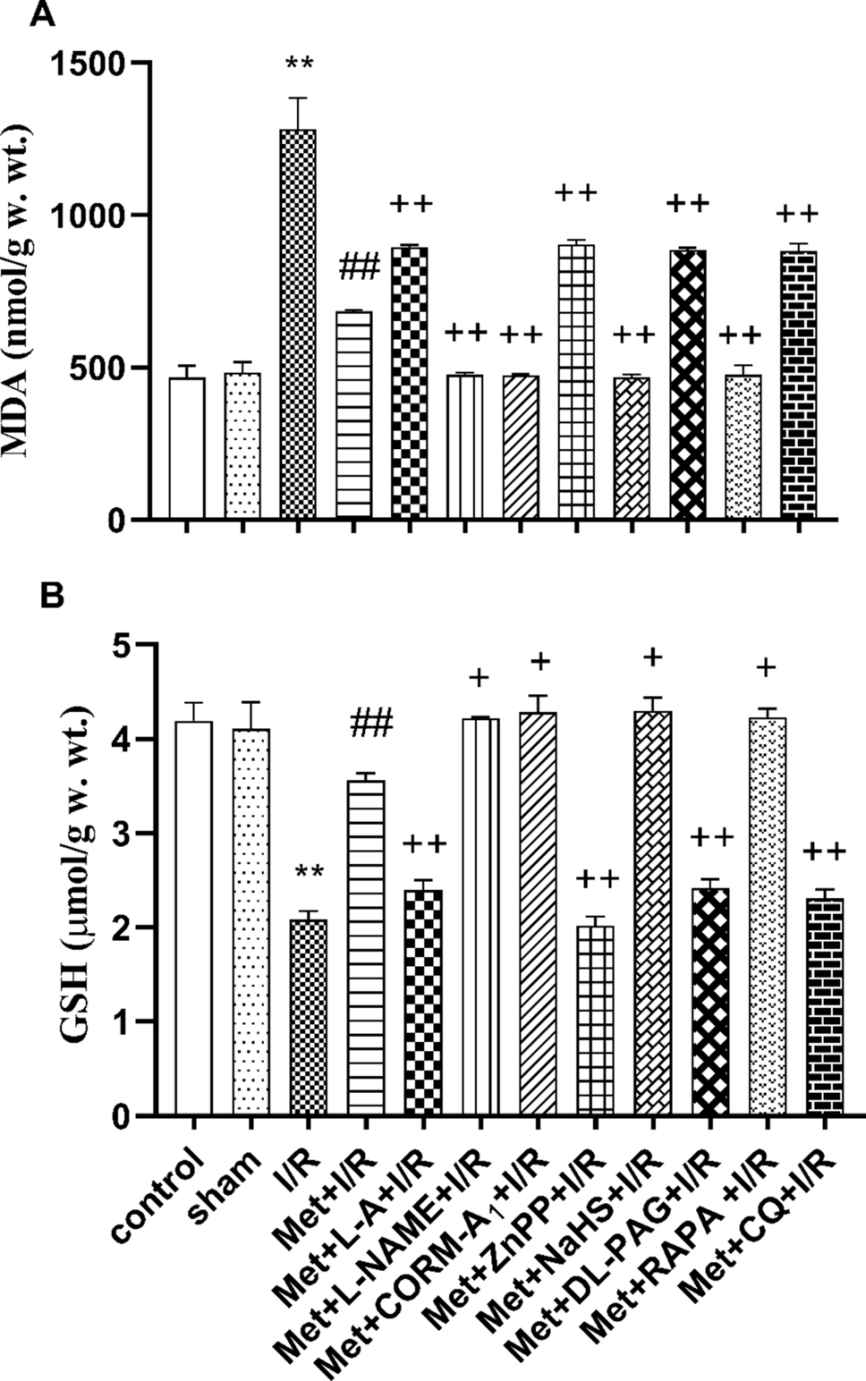
Effect of concomitant administration of nitric oxide (NO), carbon monoxide (CO), hydrogen sulfide (H_2_S), or autophagy modulators with 200 mg/kg/day metformin (Met) i.p. to rats for 6 successive days before induction of ischemia and promptly at the onset of reperfusion on the **A** malondialdehyde (MDA) and **B** intracellular reduced glutathione (GSH) levels. Animals were treated with 100 mg/kg/day L-arginine (L-A), 10 mg/kg/day L-N(G)-nitroarginine methyl ester (L-NAME), 0.1 mg/kg/day carbon monoxide releasing molecule-A_1_ (CORM-A_1_), 0.25 mg/kg/day zinc protoporphyrin (ZnPP), 3 mg/kg/day sodium hydrosulfide (NaHS), 5 mg/kg/day DL-propargylglycine (DL-PAG), 1 mg/kg/day rapamycin (RAPA), or 10 mg/kg/day chloroquine (CQ) i.p. concomitantly with Met. To induce hepatic I/R, the rats were subjected to 1 h of ischemia followed by 2 h of reperfusion. Liver tissue was collected for biochemical measurements at the end of experimental duration. Each value represents the mean ± S.E.M. of 6 observations. ^**^p< 0.01 vs. control and sham values; ^##^*p*<0.01 vs. I/R values; ^+^*p*<0.05 vs. Met+ IR values; ^++^*p*<0.01 vs. Met+I/R values

**Fig. 3:**
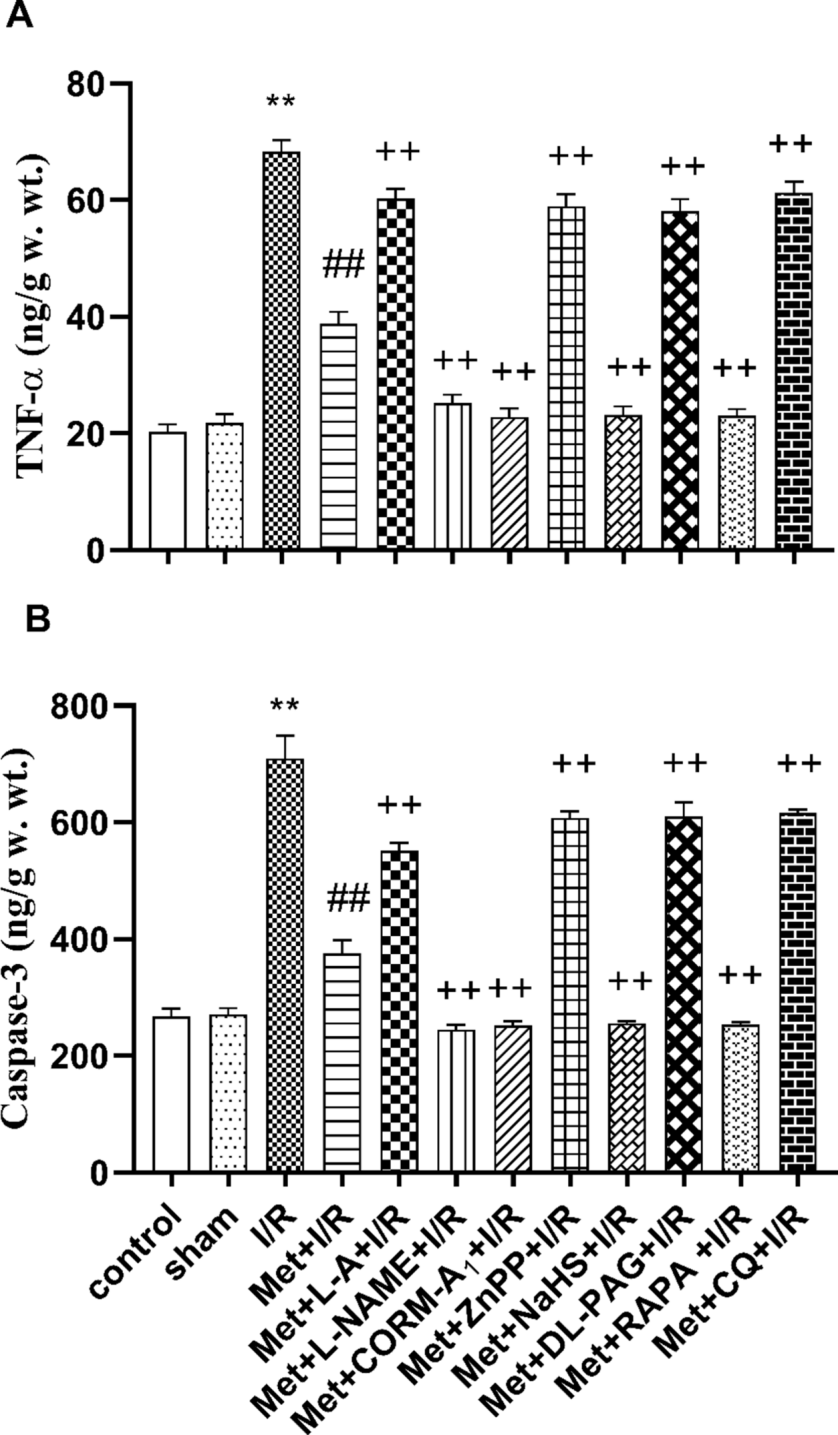
Effect of concomitant administration of nitric oxide (NO), carbon monoxide (CO), hydrogen sulfide (H_2_S) or autophagy modulators with 200 mg/kg/day metformin (Met) i.p. to rats for 6 successive days before induction of ischemia and promptly at the onset of reperfusion on the **A** tumor necrosis factor-alpha (TNF-α) and **B** caspase-3 levels. Animals were treated with 100 mg/kg/day L-arginine (L-A), 10 mg/kg/day L-N(G)-nitroarginine methyl ester (L-NAME), 0.1 mg/kg/day carbon monoxide releasing molecule-A_1_ (CORM-A_1_), 0.25 mg/kg/day zinc protoporphyrin (ZnPP), 3 mg/kg/day sodium hydrosulfide (NaHS), 5 mg/kg/day DL-propargylglycine (DL-PAG), 1 mg/kg/day rapamycin (RAPA), or 10 mg/kg/day chloroquine (CQ) i.p. concomitantly with Met. To induce hepatic I/R, the rats were subjected to 1 h of ischemia followed by 2 h of reperfusion. Liver tissue was collected for biochemical measurements at the end of experimental duration. Each value represents the mean ± S.E.M. of 6 observations. ^**^p< 0.01 vs. control and sham values; ^##^*p*<0.01 vs. I/R values; ^++^*p*<0.01 vs. Met+I/R values

Also, hepatic ischemia for 1 h followed by reperfusion for 2 h produces a significant increase in the hepatic MDA level (Fig. 2A), TNF-α level (Fig. 3A), and caspase-3 level (Fig. 3B) and produces a significant reduction in GSH level (Fig. 2B) as compared to control and sham-operated animals.

### Effect of hepatic ischemia/reperfusion (I/R) on the hepatic nitrite, heme-oxygenase-1 (HO-1) and hydrogen sulfide (H_2_S) levels

**Fig. 4:**
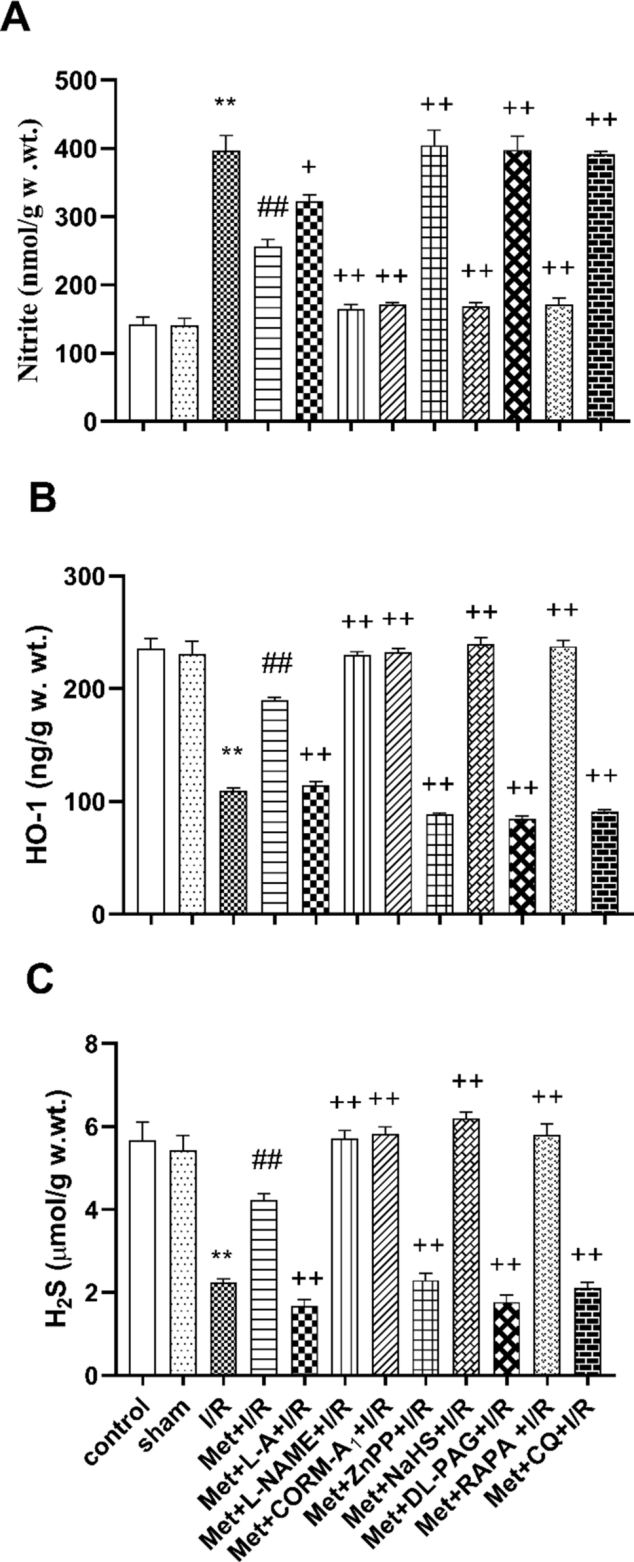
Effect of concomitant administration of nitric oxide (NO), carbon monoxide (CO), hydrogen sulfide (H_2_S), or autophagy modulators with 200 mg/kg/day metformin (Met) i.p. to rats for 6 successive days before induction of ischemia and promptly at the onset of reperfusion on the **A** nitrite, **B** heme oxygenase-1 (HO-1), and **C** hydrogen sulfide (H_2_S) levels. Animals were treated with 100 mg/kg/day L-arginine (L-A), 10 mg/kg/day L-N(G)-nitroarginine methyl ester (L-NAME), 0.1 mg/kg/day carbon monoxide releasing molecule-A_1_ (CORM-A_1_), 0.25 mg/kg/day zinc protoporphyrin (ZnPP), 3 mg/kg/day sodium hydrosulfide (NaHS), 5 mg/kg/day DL-propargylglycine (DL-PAG), 1 mg/kg/day rapamycin (RAPA), or 10 mg/kg/day chloroquine (CQ) i.p. concomitantly with Met. To induce hepatic I/R, the rats were subjected to 1 h of ischemia followed by 2 h of reperfusion. Liver tissue was collected for biochemical measurements at the end of experimental duration. Each value represents the mean ± S.E.M. of 6 observations. **p< 0.01 vs. control and sham values; ^##^*p*<0.01 vs. I/R values; ^+^*p*<0.05 vs. Met+IR values; ^++^*p*<0.01 vs. Met+I/R values

Hepatic ischemia for 1 h followed by reperfusion for 2 h significantly elevated the hepatic nitrite level (Fig. 4A) and significantly reduced the hepatic HO-1 (Fig. 4B) and H_2_S (Fig. 4C) levels in comparison to control and sham-operated animals.

Regarding all these parameters, no significant difference was found between the control and sham-operated animals.

### Effect of metformin and metformin in combination with nitric oxide (NO), carbon monoxide (CO), hydrogen sulfide (H_2_S), or autophagy modulators on the rat liver function

Administration of 200 mg/kg/day metformin i.p. for 6 successive days before induction of hepatic ischemia and promptly at the onset of reperfusion to rats significantly decreased the serum ALT (Fig. 1A) and AST (Fig. 1B) levels as compared to animals subjected to hepatic I/R.

Figure 1 shows that daily administration of 200 mg/kg/day metformin concurrently with 10 mg/kg L-N(G)-nitroarginine methyl ester (L-NAME), 0.1 mg/kg carbon monoxide releasing molecule-A_1_ (CORM-A_1_), 3 mg/kg sodium hydrosulfide (NaHS), or 1 mg/kg rapamycin (RAPA) i.p. for 6 successive days before induction of ischemia and promptly at the onset of reperfusion to rats significantly decreased the serum ALT (Fig. 1A) and AST (Fig. 1B) levels as compared to animals treated similarly with metformin only. Concurrent administration of 200 mg/kg/day metformin with 100 mg/kg/day L-arginine (L-A), 0.25 mg/kg/day zinc protoporphyrin (ZnPP), 5 mg/kg/day DL-propargylglycine (DL-PAG), or 10 mg/kg/day chloroquine (CQ) i.p. for 6 successive days before induction of ischemia and promptly at the onset of reperfusion to rats significantly increased the serum ALT (Fig. 1A) and AST (Fig. 1B) levels as compared to animals treated with metformin only.

### Effect of metformin and metformin in combination with nitric oxide (NO), carbon monoxide (CO), hydrogen sulfide (H_2_S), or autophagy modulators on the hepatic levels of biochemical parameters of rats subjected to hepatic ischemia/reperfusion (I/R)

Administration of 200 mg/kg/day metformin i.p. for 6 successive days before induction of ischemia and promptly at the onset of reperfusion to rats significantly reduced the hepatic MDA level (Fig. 2A) and elevated the hepatic intracellular GSH level (Fig. 2B) in contrast to animals subjected to hepatic I/R.

Daily administration of 200 mg/kg metformin concurrently with 10 mg/kg L-NAME, 0.1 mg/kg CORM-A_1,_ 3 mg/kg NaHS, or 1mg/kg RAPA i.p. for 6 successive days before induction of ischemia and promptly at the onset of reperfusion to rats produced a significant reduction in the hepatic MDA level (Fig. 2A) and a significant increase in the hepatic GSH level (Fig. 2B) as compared to animals treated similarly with metformin only. Concomitant administration of 200 mg/kg/day metformin with 100 mg/kg/day L-A, 0.25 mg/kg/day ZnPP, 5 mg/kg/day DL-PAG, or 10 mg/kg/day CQ i.p. for 6 successive days before induction of ischemia and promptly at the onset of reperfusion to rats significantly increased the hepatic MDA level (Fig. 2A) and decreased the hepatic GSH level (Fig. 2B) in comparison to animals treated with metformin only.

Figure 3 illustrates that administration of 200 mg/kg/day metformin i.p. for 6 successive days before induction of ischemia and promptly at the onset of reperfusion to rats significantly lowered the hepatic TNF-α (Fig. 3A) and caspase-3 (Fig. 3B) levels as compared to animals subjected to hepatic I/R.

Daily administration of 200 mg/kg metformin concurrently with 10 mg/kg L-NAME, 0.1 mg/kg CORM-A_1_, 3 mg/kg NaHS, or 1mg/kg RAPA i.p. for 6 successive days before induction of ischemia and promptly at the onset of reperfusion to rats produced a significant reduction in the hepatic TNF-α (Fig. 3A) and caspase-3 (Fig. 3B) levels as compared to animals treated similarly with metformin only. In comparison to animals treated with metformin only, concomitant administration of 200 mg/kg/day metformin with 100 mg/kg L-A, 0.25 mg/kg/day ZnPP, 5 mg/kg DL-PAG, or 10 mg/kg/day CQ i.p. for 6 successive days before induction of ischemia and promptly at the onset of reperfusion to rats produced a significant increase in the hepatic TNF-α (Fig. 3A) and caspase-3 (Fig. 3B) levels.

### Effect of metformin and metformin in combination with nitric oxide (NO), carbon monoxide (CO), hydrogen sulfide (H_2_S), or autophagy modulators on the hepatic nitrite, heme-oxygenase-1, and hydrogen sulfide levels

Administration of 200 mg/kg/day metformin i.p. for 6 successive days before induction of ischemia and promptly at the onset of reperfusion to rats significantly decreased the hepatic nitrite level (Fig. 4A) and increased the hepatic HO-1 (Fig. 4B) and H_2_S (Fig. 4C) levels as compared to animals subjected to hepatic I/R.

Daily administration of 200 mg/kg/day metformin concurrently with 10 mg/kg L-NAME, 0.1 mg/kg CORM-A_1_, 3 mg/ kg NaHS or 1mg/kg RAPA i.p. for 6 successive days before induction of ischemia and promptly at the onset of reperfusion to rats significantly reduced the hepatic nitrite level (Fig. 4A) and produced a significant increase in the hepatic HO-1 (Fig. 4B) and H_2_S (Fig. 4C) levels as compared to animals treated similarly with metformin only. Co-administration of 200 mg/kg/day metformin with 100 mg/kg/day L-A, 0.25 mg/kg/day ZnPP, 5 mg/kg/day DL-PAG or 10 mg/kg/day CQ i.p. for 6 successive days before induction of ischemia and promptly at the onset of reperfusion to rats significantly raised the hepatic nitrite level (Fig. 4A) and significantly lowered the hepatic HO-1 (Fig. 4B) and H_2_S (Fig. 4C) levels as compared to animals treated with metformin only.

### The histopathological examination of the liver tissue

**Fig. 5:**
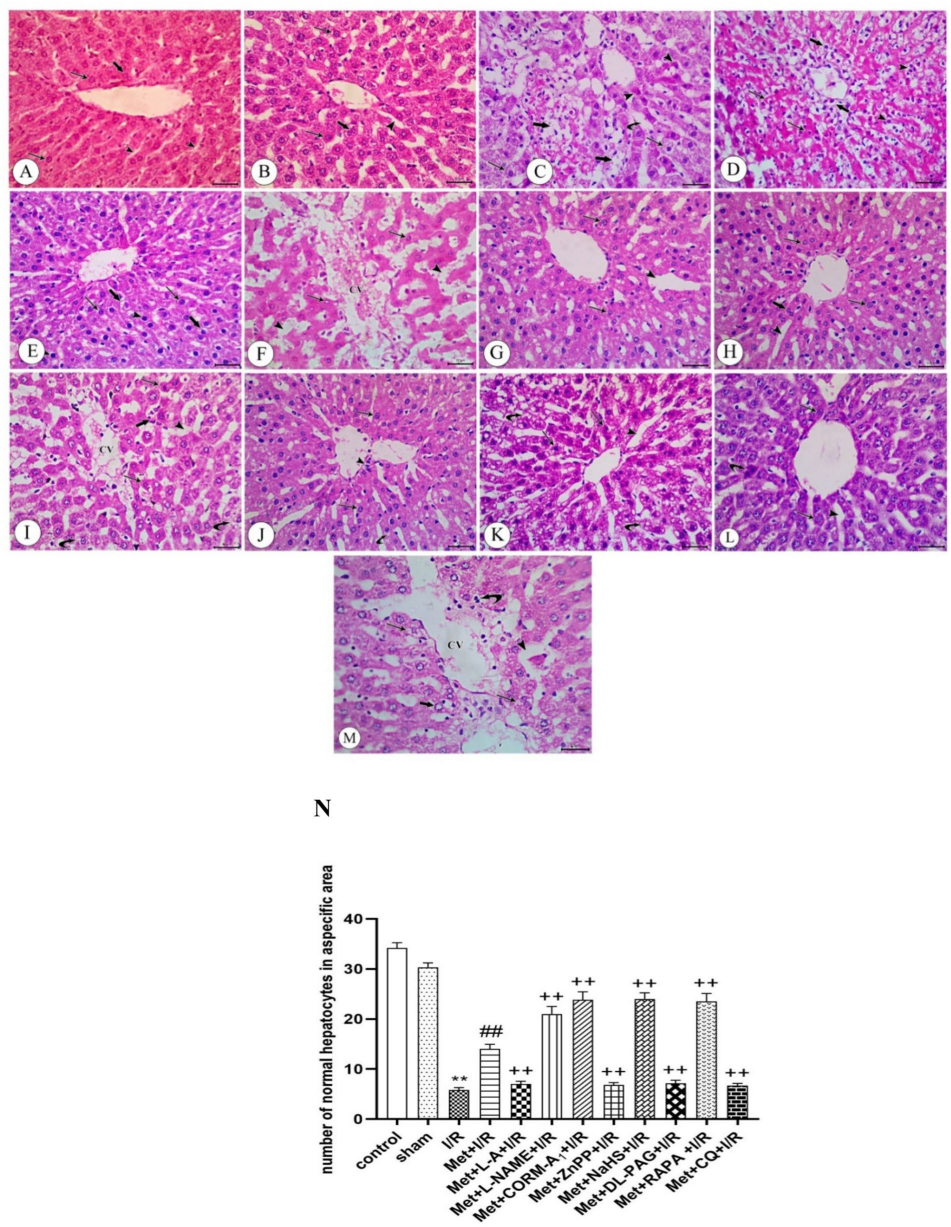
photomicrographs of representative liver sections from **A** control and **B** sham rats: showing normal hepatic architecture with hepatocytes arranged in branching and anastomosing cords separated by blood sinusoids (arrow heads). Hepatocytes have central rounded vesicular nuclei and acidophilic cytoplasm (arrows). Few cells appear binucleated (thick arrow). **C** I/R rats showing loss of normal architecture of hepatocytes. Most of hepatocytes appear degenerated with cytoplasmic vacuolations (arrows); other appear with deeply stained cytoplasm and deeply stained irregular nuclei (arrow head). Some hepatocytes are ballooned with remnants of cytoplasm and the nuclei either deeply stained or absent and the fused cells with no cell boundaries (thick arrows). Dilated blood sinusoids in between hepatic cords are also detected (curved arrow). **D** I/R rats showing disrupted liver architecture with inflammatory cell infiltration (arrow head). Most of hepatocytes appear with deeply stained acidophilic cytoplasm and lost their boundaries with deeply stained nuclei (arrows). Other appear vacuolated especially around central vein (thick arrow). **E** I/R rats were treated with Met showing improvement of the general architecture of liver, most of hepatocytes appear with acidophilic cytoplasm and central vesicular nuclei (arrows), but others appear with deeply stained nuclei (arrow heads). Some hepatocytes show cytoplasmic vacuolations (thick arrows). **F** I/R rats treated with Met+L-A showing marked disruption in the normal architecture of the liver with distorted congested central vein (CV). Most of hepatocytes appear with deeply stained cytoplasm and irregular shaped nuclei (arrows). Dilated, disturbed and congested blood sinusoid are also detected in between hepatocytes (arrow head) **G** I/R rats treated with Met+L-NAME showing marked improvement in histopathological changes of hepatocytes with dilated blood sinusoids in between (arrow head). Most of hepatocytes appear with rounded vesicular nuclei and acidophilic cytoplasm with minimal vacuolation (arrows) **H** I/R rats treated with Met+CORM-A_1_ showing an apparent normal architecture of liver tissue. Hepatocytes arranged in cords separating by blood sinusoids (arrow head). Most of hepatocytes appear with rounded vesicular nuclei and acidophilic cytoplasm (arrows). Some cells appear binucleated (thick arrow) **I** I/R rats treated with Met+ZnPP showing loss of architecture with irregular dilated blood sinusoids (arrow head). The central vein appears irregularly (CV) distorted with inflammatory cell infiltration (thick arrow). Some of hepatocytes appear with deeply stained cytoplasm and irregular shaped nuclei(arrows), others appear with vacuolated cytoplasm (curved arrows) **J** I/R rats treated with Met+NaHS showing moderate improvement in histological appearance, some of hepatocytes appear with acidophilic cytoplasm and central vesicular nuclei (arrows). Other hepatocytes show minimal vacuolated cytoplasm (curved arrows). Minimal inflammatory cell infiltration is also noticed especially near the central vein (arrow head). **K** I/R rats treated with Met+DL-PAG showing disturbed irregular pattern of hepatic cords separated by dilated blood sinusoids (arrow head). Most of hepatocytes appear with marked intracellular vacuolations (curved arrows). Other hepatocytes appear with deeply stained cytoplasm and irregular nuclei (arrows). **L** I/R rats treated with Met+RAPA showing marked amelioration in hepatic changes with mild hepatic blood sinusoidal dilatation (arrow head). Most of hepatocytes appear with vesicular nuclei (arrows); few appear with deeply stained cytoplasm and irregular nuclei (curved arrow). **M** I/R rats treated with Met+CQ showing severe dilatation and disruption of the central vein (CV) with cellular inflammatory infiltration (curved arrow). Hepatocytes appear with vacuolar degeneration (arrows); dilated irregular blood sinusoids are also detected (arrow head). Some nuclei appear with marginated chromatin (thick arrow) (H&E (x400)). **N** A histogram showing the number of normal hepatocytes in a specific area

**Fig. 6:**
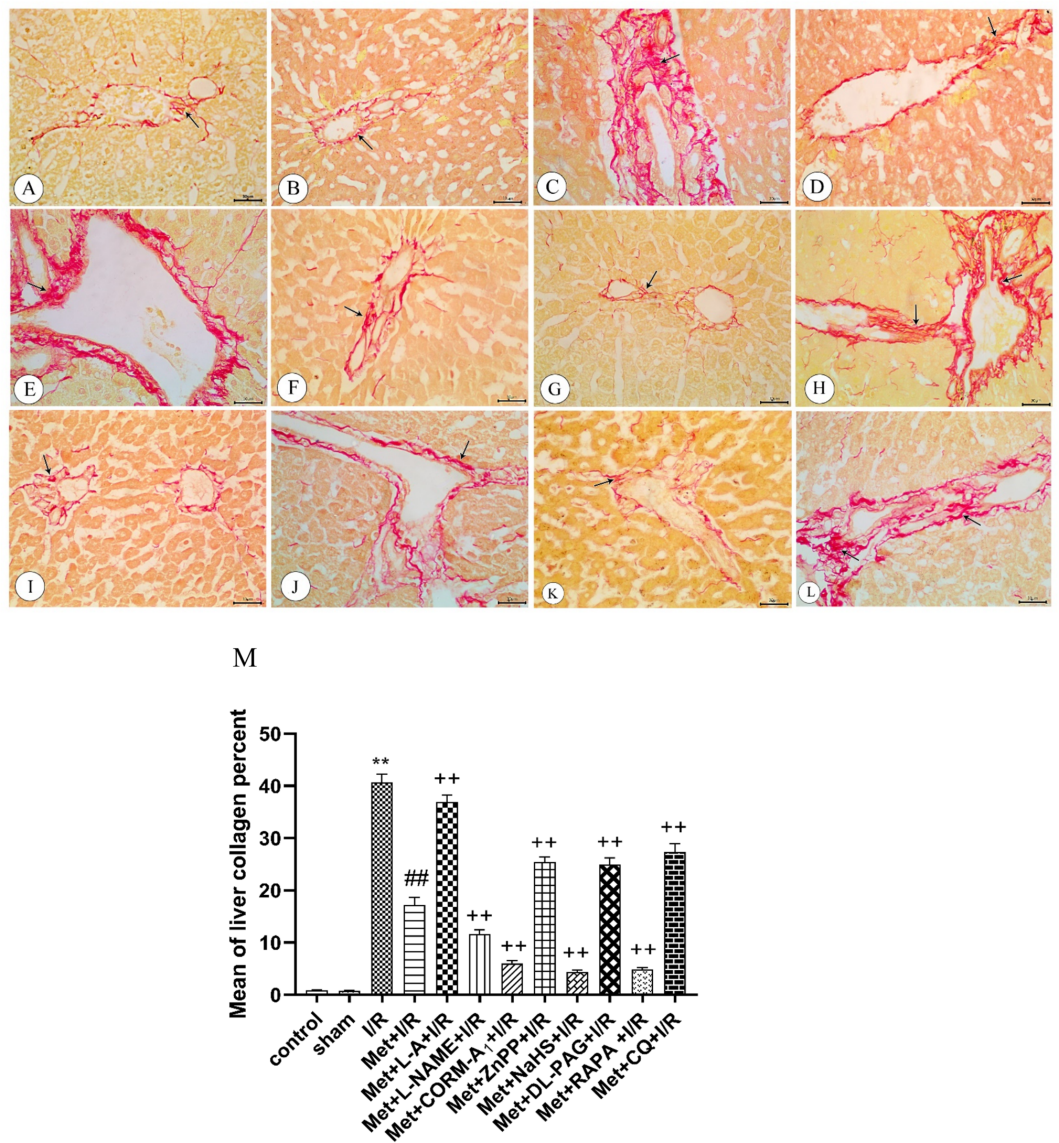
photomicrographs of representative liver sections from **A** control and **B** sham rats: showing minimal amount of collagen fibers around the portal area (arrow). **C** I/R rats showing apparent increase in the collagen fibers around the portal area (arrow). **D** I/R rats treated with Met showing apparent decrease in the collagen fibers around the portal area (arrow). **E** I/R rats treated with Met+L-A showing apparent increase in the collagen fibers around the portal area (arrow). **F** I/R rats treated with Met+L-NAME showing apparent decrease in the collagen fibers around the portal area (arrow). **G** I/R rats treated with Met +CORM-A_1_ showing apparent decrease in the collagen fibers around the portal area (arrow). **H** I/R rats treated with Met+ZnPP showing apparent increase in the collagen fibers around the portal area (arrows). **I** I/R rats treated with Met+NaHS showing apparent decrease in the collagen fibers around the portal area (arrow). **J** I/R rats treated with Met+DL-PAG showing apparent increase in the collagen fibers around the portal area (arrow). **K** I/R rats treated with Met+RAPA showing apparent decrease in the collagen fibers around the portal area (arrow). **L** I/R rats treated with Met+CQ showing apparent increase in the collagen fibers around the portal area (arrows). **M** A histogram showing estimation of mean of liver collagen percent

The histopathological examination of the liver tissue obtained from control and sham rats showed normal hepatic architecture with hepatocytes arranged in branching and anastomosing cords separated by blood sinusoids. Hepatocytes contain centrally rounded vesicular nuclei and acidophilic cytoplasm. Few cells appear binucleated (Fig. 5A and B). Sirius red-stained sections of the both groups revealed a minimum amount of collagen fibers around the portal area (Fig. 6, A&B)

As seen in Fig. 5C and D liver tissue obtained from rats subjected to 1 h of ischemia followed by reperfusion for 2 h (I/R) showed loss of normal architecture of hepatocytes with dilated blood sinusoids in-between hepatic cords. Most of hepatocytes appear degenerated with cytoplasmic vacuolations especially around central vein, others appear with deeply stained cytoplasm and deeply stained irregular nuclei. Some hepatocytes are ballooned with cytoplasm remnants and the nuclei that are either deeply stained or absent and the fused cells with no cell boundaries. Additionally, inflammatory cell infiltration was also detected. Furthermore, examination of Sirius red-stained sections from the liver revealed an apparent increase of the collagen fibers around the portal area (Fig. 6C).

Administration of 200 mg/kg/day metformin i.p. for 6 successive days before induction of ischemia and promptly at the onset of reperfusion to rats produced improvement of the general architecture of liver. Most of hepatocytes appear with acidophilic cytoplasm and central vesicular nuclei but others appear with deeply stained nuclei. Some hepatocytes show cytoplasmic vacuolations (Fig. 5E). Moreover, Sirius red-stained sections showed a considerable reduction in the collagen fibers around the portal area (Fig. 6D)

Daily administration of 200 mg/kg metformin concurrently with 100 mg/kg L-A i.p. for 6 successive days before induction of ischemia and promptly at the onset of reperfusion to rats produced marked disruption in the normal architecture of the liver with distorted congested central vein. Most of hepatocytes appear with deeply stained cytoplasm and irregular shaped nuclei. Dilated, disturbed and congested blood sinusoids were also detected in between hepatocytes (Fig. 5F). Sirius red-stained sections revealed a noticeable increase in the collagen fibers around the portal area (Fig. 6E).

Concurrent administration of 200 mg/kg/day metformin with 10 mg/kg/day L-NAME i.p. for 6 successive days before induction of ischemia and promptly at the onset of reperfusion to rats produced marked improvement in histopathological changes of hepatocytes with dilated blood sinusoids in between. Most of hepatocytes appear with rounded vesicular nuclei and acidophilic cytoplasm with minimal vacuolation (Fig. 5G). Sirius red-stained sections showed an apparent reduction in the collagen fibers around the portal area (Fig. 6F)

Concurrent administration of 200 mg/kg/day metformin with 0.1 mg/kg/day CORM-A_1_ i.p. for 6 successive days before induction of ischemia and promptly at the onset of reperfusion to rats produced an apparent normal architecture of liver tissue. Hepatocytes arranged in cords separating by blood sinusoids. Most of hepatocytes appear with rounded vesicular nuclei and acidophilic cytoplasm. Some cells appear binucleated (Fig. 5H). Furthermore, Sirius red-stained sections revealed a considerable decrease in the collagen fibers around the portal area (Fig. 6G).

Administration of 200 mg/kg/day metformin concomitantly with 0.25 mg/kg/day ZnPP i.p. for 6 successive days before induction of ischemia and promptly at the onset of reperfusion to rats showed loss of architecture with irregular dilated blood sinusoids. The central vein appeared irregularly distorted with inflammatory cell infiltration. Some of hepatocytes appear with deeply stained cytoplasm and irregular shaped nuclei, others appear with vacuolated cytoplasm (Fig. 5I). Moreover, Sirius red-stained sections revealed a noticeable increase in the collagen fibers around the portal area (Fig. 6H).

Daily administration of 200 mg/kg metformin concurrently with 3 mg/kg NaHS i.p. for 6 successive days before induction of ischemia and promptly at the onset of reperfusion to rats produced moderate improvement in histological appearance, some of hepatocytes with acidophilic cytoplasm and central vesicular nuclei. Other hepatocytes show minimal vacuolated cytoplasm. Minimal inflammatory cell infiltration is also noticed especially near the central vein (Fig. 5J). Sirius red-stained sections showed a considerable decrease in the collagen fibers around the portal area (Fig. 6I).

Concurrent administration of 200 mg/kg/day metformin with 5 mg/kg/day DL-PAG i.p. for 6 successive days before induction of ischemia and promptly at the onset of reperfusion to rats showed disturbed irregular pattern of hepatic cords separated by dilated blood sinusoids. Most of hepatocytes appear with marked intracellular vacuolations. Other hepatocytes appear with deeply stained cytoplasm and irregular nuclei (Fig. 5K). Sirius red-stained sections revealed a considerable increase in the collagen fibers around the portal area (Fig. 6J).

Daily administration of 200 mg/kg metformin concurrently with 1mg/kg RAPA i.p. for 6 successive days before induction of ischemia and promptly at the onset of reperfusion to rats produced marked amelioration in hepatic changes with mild hepatic blood sinusoidal dilatation. Most of hepatocytes appear with vesicular nuclei, few appear with deeply stained cytoplasm and irregular nuclei (Fig. 5L). Also, Sirius red-stained sections showed an apparent decline in the collagen fibers around the portal area (Fig. 6K).

Concurrent administration of 200 mg/kg/day metformin with 10 mg/kg/day CQ i.p. for 6 successive days before induction of ischemia and promptly at the onset of reperfusion to rats showed severe dilatation and disruption of the central vein with cellular inflammatory infiltration. Hepatocytes appear with vacuolar degeneration, dilated irregular blood sinusoids are also detected. Some nuclei appear with marginated chromatin (Fig. 5M). Furthermore, Sirius red-stained sections revealed a marked increase in the collagen fibers around the portal area (Fig. 6L).

### Immunohistochemical analysis

**Fig. 7:**
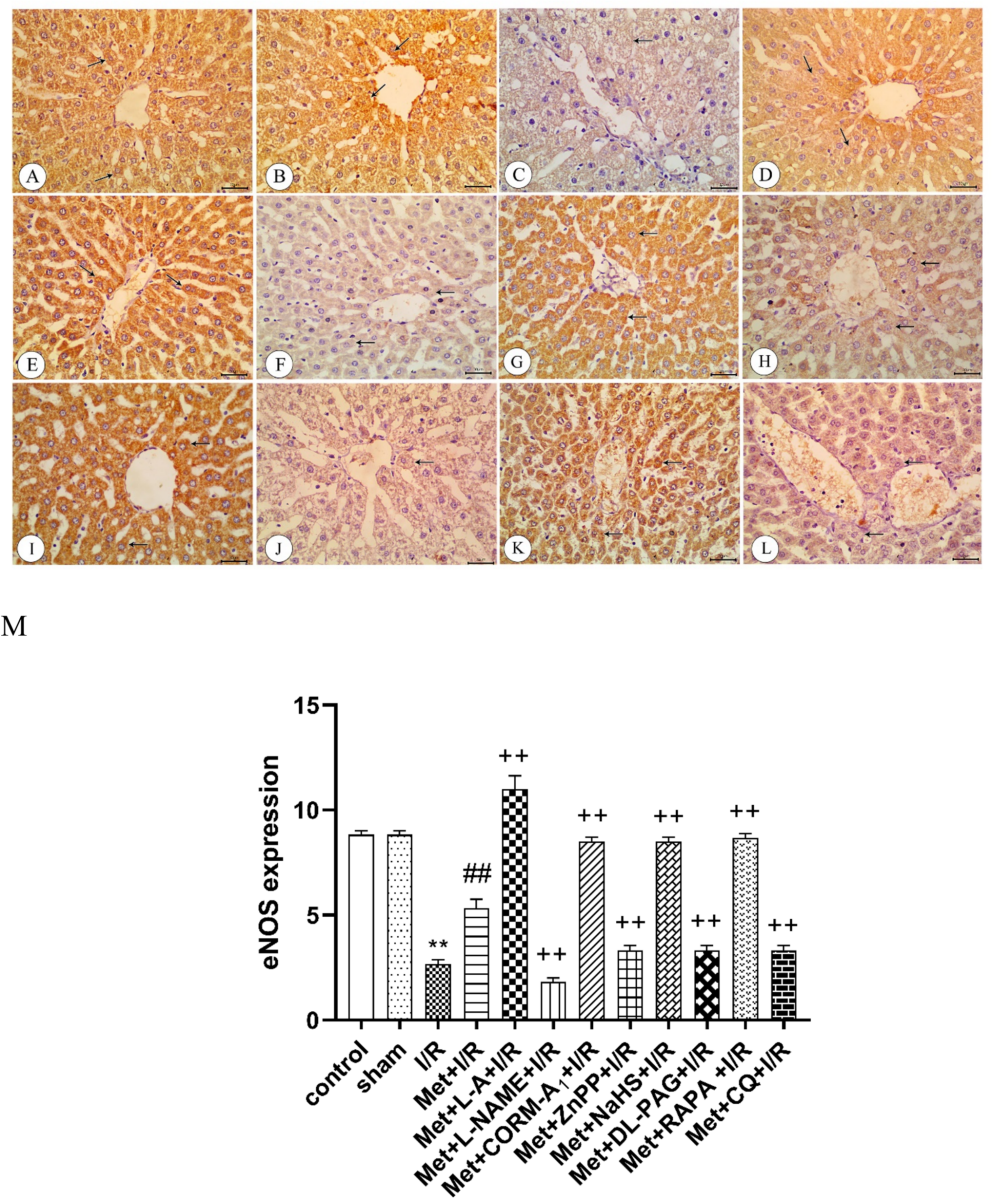
Immunohistochemistry of endothelial nitric oxide synthase (eNOS) in liver sections from **A** control rats showing strong expression of eNOS (arrows), **B** sham rats showing strong expression of eNOS (arrows), **C** I/R rats showing weak expression of eNOS (arrow), **D** I/R rats treated with Met showing moderate expression of eNOS (arrows), **E **I/R rats treated with Met+L-A showing strong expression of eNOS (arrows), **F** I/R rats treated with Met+L-NAME showing weak expression of eNOS (arrows), **G** I/R rats treated with Met+CORM-A_1_ showing strong expression of eNOS(arrows), **H** I/R rats treated with Met+ZnPP showing weak expression of eNOS, **I** I/R rats treated with Met+NaHS showing strong expression of eNOS, **J** I/R rats treated with Met+DL-PAG showing weak expression of eNOS, **K** I/R rats treated with Met+RAPA showing strong expression of eNOS, and **L** I/R rats treated with Met+CQ showing weak expression of eNOS. **M** Protein expression of eNOS in liver tissues of rats subjected to 1 h of ischemia followed by 2 h of reperfusion treated with Met, Met +L-A, Met+L-NAME, Met+CORM-A_1_, Met+ZnPP, Met+NaHS, Met+DL-PAG, Met+RAPA, and Met+CQ and. Each value represents the mean ± S.E.M. of 6 observations. ^**^Significant difference at *p*<0.01 vs. control and sham values. ^##^Significant difference at *p*<0.01 vs. I/R values. ^++^Significant difference at *p*<0.01 vs. Met +I/R values

**Fig. 8:**
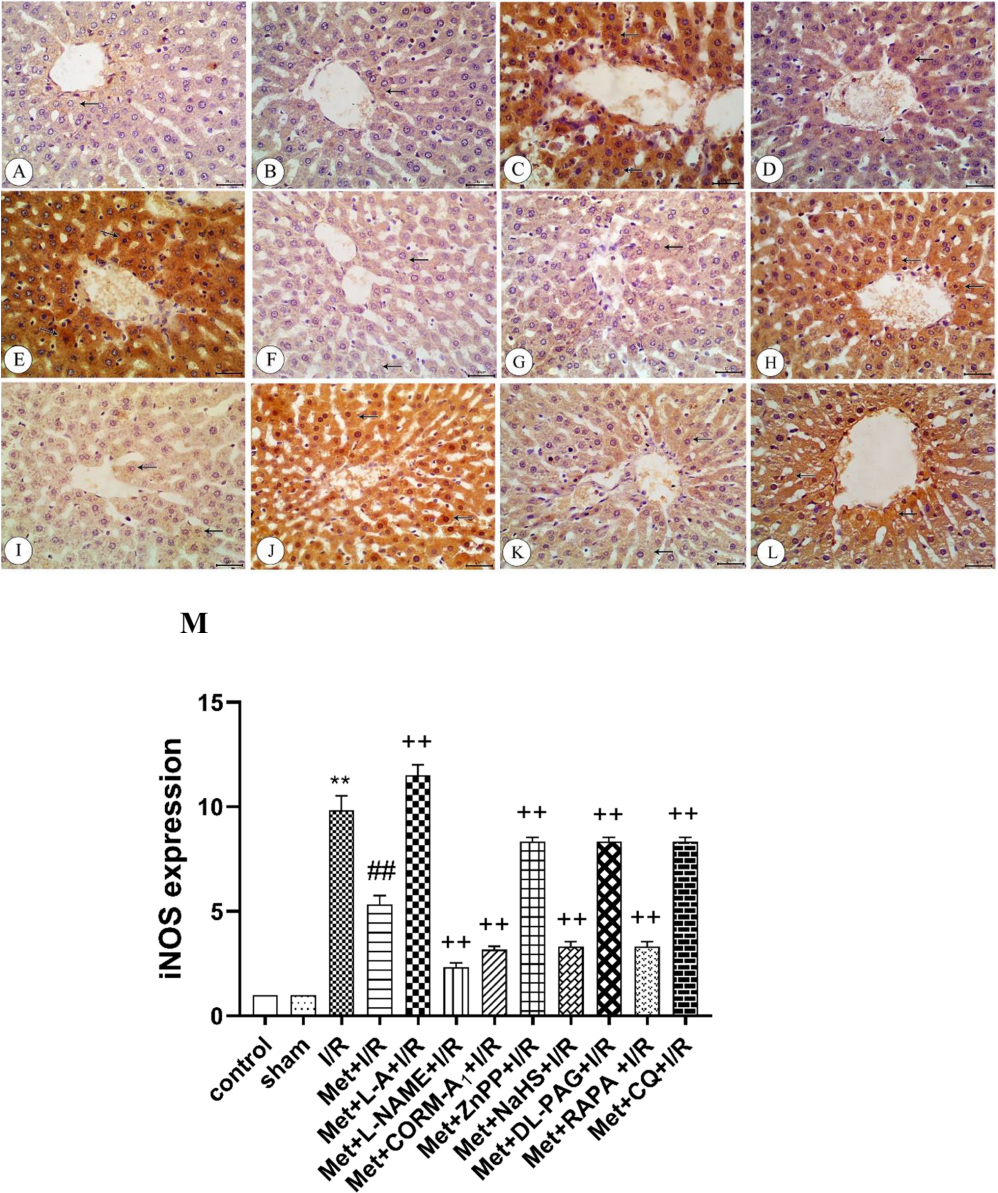
Immunohistochemistry of inducible nitric oxide synthase (iNOS) in liver tissues from **A** Control rats showing weak expression of iNOS (arrow), **B** sham rats showing weak expression of iNOS (arrow), **C** I/R rats showing strong expression of iNOS, **D** I/R rats treated with Met showing moderate expression of iNOS (arrows), **E** I/R rats treated with Met+L-A showing strong expression of iNOS (arrows), **F** I/R rats treated with Met+L-NAME showing weak expression of iNOS (arrows), **G** I/R rats treated with Met+CORM-A_1_ showing weak expression of iNOS (arrow), **H** I/R rats treated with Met+ZnPP showing strong expression of iNOS (arrows), **I** I/R rats treated with Met+NaHS showing weak expression of iNOS (arrows), **J** I/R rats treated with Met+DL-PAG showing strong expression of iNOS (arrows), **K** I/R rats treated with Met+RAPA showing weak expression of iNOS (arrows), and **L** I/R rats treated with Met+CQ showing strong expression of iNOS (arrows). **M** Protein expression of iNOS in liver tissues of rats subjected to 1 h of ischemia followed by 2 h of reperfusion treated with Met, Met+L-A, Met+L-NAME, Met+CORM-A_1_, Met+ZnPP, Met+NaHS, Met+DL-PAG, Met+RAPA, and Met+CQ. Each value represents the mean ± S.E.M. of 6 observations. ^**^Significant difference at *p*<0.01 vs. control and sham values. ^##^Significant difference at *p*<0.01 vs. I/R values. ^++^Significant difference at *p*<0.01 vs. Met+I/R values

**Fig. 9:**
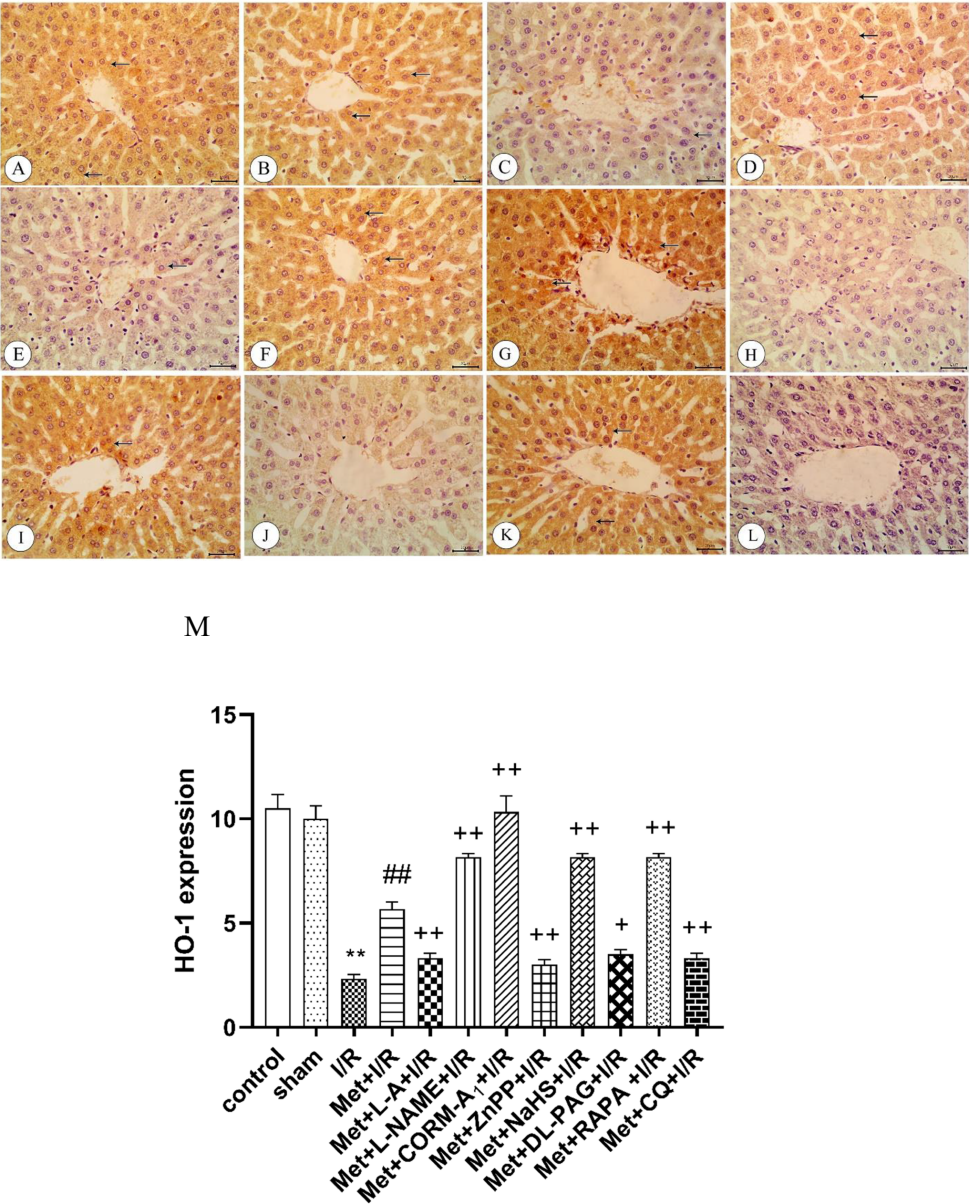
Immunohistochemistry of heme oxygenase-1 (HO-1) in liver tissues from **A** control rats showing strong expression of HO-1(arrows), **B** sham rats showing strong expression of HO-1(arrows), **C** I/R rats showing weak expression of HO-1(arrow), **D** I/R rats treated with Met showing moderate expression of HO-1(arrows), **E** I/R rats treated with Met+L-A showing weak expression of HO-1(arrow), **F** I/R rats treated with Met+L-NAME showing strong expression of HO-1(arrows), **G** I/R rats treated with Met+CORM-A_1_ showing strong expression of HO-1(arrows), **H** I/R rats treated with Met+ZnPP showing weak expression of HO-1, **I** I/R rats treated with Met+NaHS showing strong expression of HO-1(arrow), **J** I/R rats treated with Met+DL-PAG showing weak expression of HO-1, **K** I/R rats treated with Met+RAPA showing strong expression of HO-1(arrows), and **L** I/R rats treated with Met+CQ showing weak expression of HO-1. **M** Protein expression of HO-1 in liver tissues of rats subjected to 1 h of ischemia followed by 2 h of reperfusion treated with Met, Met+L-A, Met+L-NAME, Met+CORM-A_1_, Met+ZnPP, Met+NaHS, Met+DL-PAG, Met+RAPA, and Met+CQ. Each value represents the mean ± S.E.M. of 6 observations. ^**^Significant difference at *p*<0.01 vs. control and sham values. ^##^Significant difference at *p*<0.01 vs. I/R values. ^+^Significant difference at *p*<0.05 vs. Met+I/R values. ^++^Significant difference at *p*<0.01 vs. Met +I/R values

**Fig. 10:**
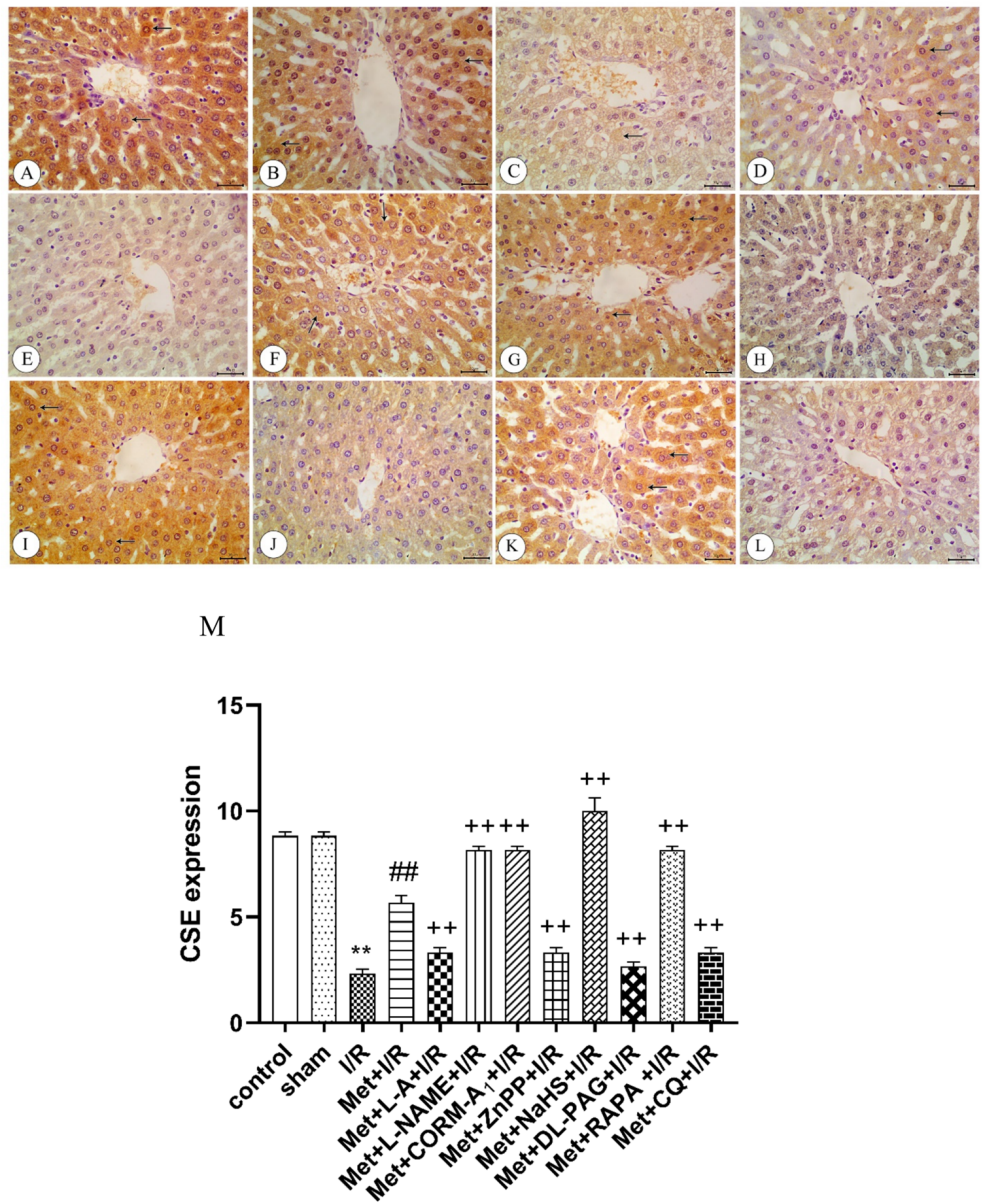
Immunohistochemistry of cystathionine-γ-lyase (CSE) in liver tissues from **A** control rats showing strong expression of CSE (arrows), **B** sham rats showing strong expression of CSE (arrows), **C** I/R rats showing weak expression of CSE (arrow), **D** I/R rats treated with Met showing moderate expression of CSE (arrows), **E** I/R rats treated with Met+L-A showing weak expression of CSE, **F** I/R rats treated with Met+L-NAME showing strong expression of CSE (arrows), **G** I/R rats treated with Met+CORM-A_1_ showing strong expression of CSE (arrows), **H** I/R rats treated with Met+ZnPP showing weak expression of CSE, **I** I/R rats treated with Met+NaHS showing strong expression of CSE (arrows), **J** I/R rats treated with Met+DL-PAG showing weak expression of CSE, **K** I/R rats treated with Met+RAPA showing strong expression of CSE (arrows), and **L** I/R rats treated with Met+CQ showing weak expression of CSE. **M** Protein expression of CSE in liver tissues of rats subjected to 1 h of ischemia followed by 2 h of reperfusion treated with Met, Met +L-A, Met +L-NAME, Met +CORM-A_1_, Met +ZnPP, Met +NaHS, Met +DL-PAG, Met+RAPA, and Met+CQ. Each value represents the mean ± S.E.M. of 6 observations. ^**^Significant difference at *p*<0.01 vs. control and sham values. ^##^Significant difference at *p*<0.01 vs. I/R values. ^++^Significant difference at *p*<0.01 vs. Met+I/R values

**Fig. 11:**
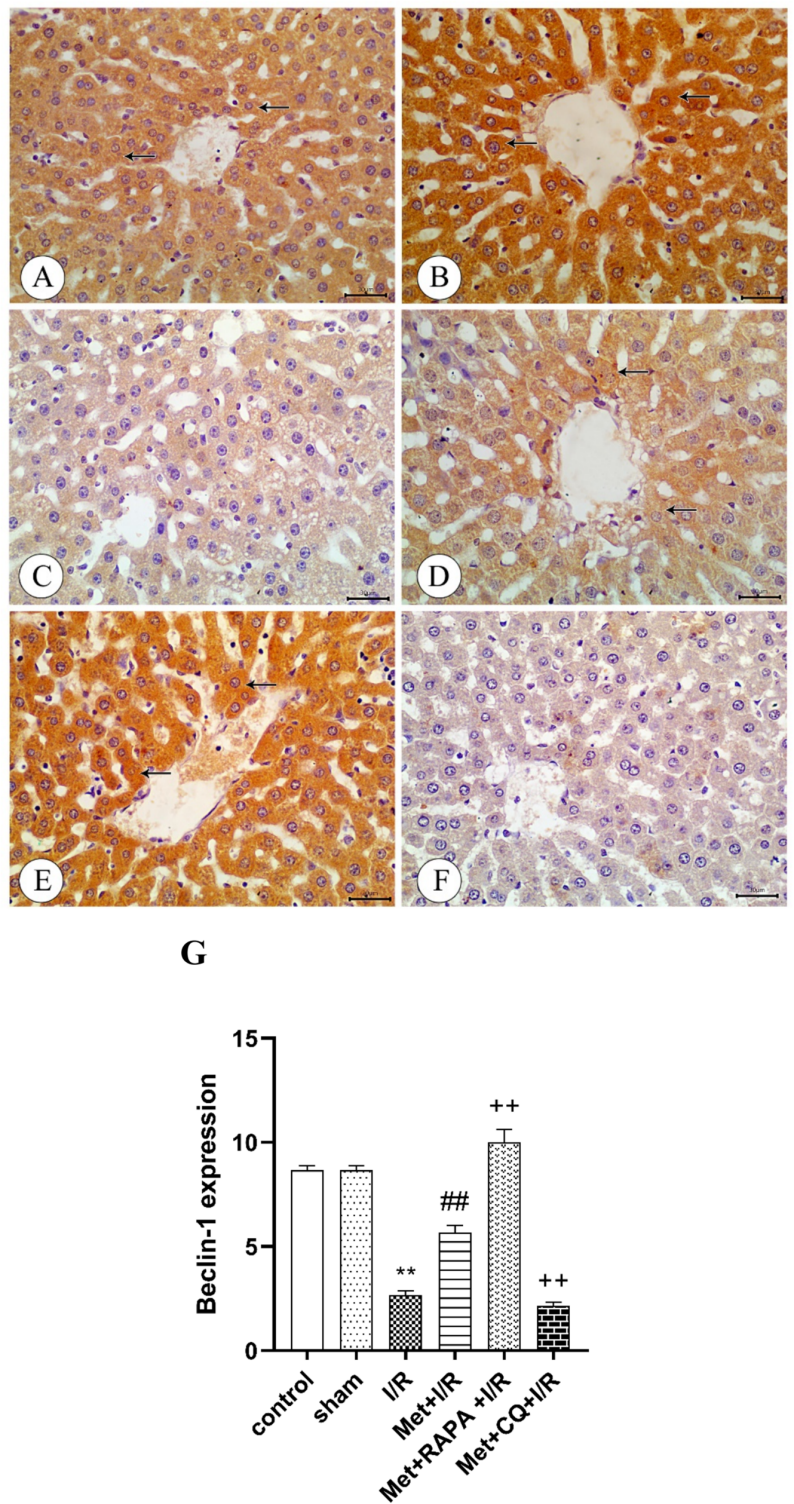
Immunohistochemistry of Beclin-1 in liver tissues from **A** control rats showing strong expression of Beclin-1, **B** sham rats showing strong expression of Beclin-1, **C** I/R rats showing weak expression of Beclin-1, **D** I/R rats treated with Met showing moderate expression of Beclin-1, **E** I/R rats treated with Met+RAPA showing strong expression of Beclin-1, and **F** I/R rats treated with Met+CQ showing weak expression of Beclin-1. **G** Protein expression of Beclin-1 in liver tissues of rats subjected to 1 h of ischemia followed by 2 h of reperfusion treated with Met, Met+RAPA, and Met+CQ. Each value represents the mean ± S.E.M. of 6 observations. ^**^Significant difference at *p*< 0.01 vs. control and sham values. ^##^Significant difference at *p*<0.01 vs. I/R values. ^++^Significant difference at *p*<0.01 vs. Met. +I/R values

The immunohistochemical examination of the liver tissue taken from rats subjected to hepatic ischemia for 1 h followed by reperfusion for 2 h (I/R) showed a decrease in the eNOS (Fig. 7), HO-1 (Fig. 9), CSE (Fig. 10) and Beclin-1 (Fig. 11) protein expression in the liver tissue in comparison to control and sham rats. The protein expression of iNOS (Fig. 8) was increased in these rats.

Administration of 200 mg/kg/day metformin i.p. for 6 successive days before induction of ischemia and promptly at the onset of reperfusion to rats enhanced eNOS (Fig. 7), HO-1 (Fig. 9), CSE (Fig. 10), and Beclin-1 (Fig. 11) protein expressions. The same treatment reduced iNOS protein expression (Fig. 8) in the liver tissue.

Daily administration of 200 mg/kg metformin concurrently with 100 mg/kg L-A i.p. for 6 successive days before induction of ischemia and promptly at the onset of reperfusion to rats elevated eNOS (Fig. 7) and iNOS (Fig. 8) protein expressions in the liver tissue. However, HO-1 (Fig. 9) and CSE (Fig. 10) protein expressions were reduced in the liver tissue. Similar treatment with metformin in combination with 10 mg/kg/day L-NAME i.p. reduced eNOS (Fig. 7) and iNOS (Fig. 8) protein expressions in the liver tissue. On the other hand, this combination increased metformin’s stimulatory effect of on the protein expressions of HO-1 (Fig. 9) and CSE (Fig. 10).

Concurrent administration of 200 mg/kg/day metformin with 0.1 mg/kg/day CORM-A_1_ i.p. for 6 successive days before induction of ischemia and promptly at the onset of reperfusion to rats elevated eNOS (Fig. 7), HO-1 (Fig. 9), and CSE (Fig. 10) protein expressions in the liver tissue. The same treatment produced a significant decrease in iNOS protein expression (Fig. 8) in the liver tissue. Similar daily administration of a combination of metformin and 0.25 mg/kg ZnPP i.p. to rats decreased eNOS (Fig. 7), HO-1 (Fig. 9), and CSE (Fig. 10) protein expressions and increased iNOS protein expression (Fig. 8) in the liver tissue.

Daily administration of 200 mg/kg metformin concurrently with 3 mg/kg NaHS i.p. for 6 successive days before induction of ischemia and promptly at the onset of reperfusion to rats increased the stimulatory effect of metformin on eNOS (Fig. 7), HO-1 (Fig. 9), and CSE (Fig. 10) protein expressions in the liver tissue. The same treatment produced a significant decrease in iNOS protein expression (Fig. 8). Similar daily treatment of rats with a combination of metformin and 5 mg/kg DL-PAG i.p. showed reduction in eNOS (Fig. 7), HO-1 (Fig. 9), and CSE (Fig. 10) protein expressions in the liver tissue. The same combination increased iNOS protein expression (Fig. 8) in the liver tissue.

Daily administration of 200 mg/kg metformin concurrently with 1mg/kg RAPA i.p. for 6 successive days before induction of ischemia and promptly at the onset of reperfusion to rats raised the stimulatory effect of metformin on eNOS (Fig. 7), HO-1 (Fig. 9), CSE (Fig. 10), and Beclin-1 (Fig. 11) protein expressions in the liver tissue. The same treatment produced a significant decrease in iNOS protein expression (Fig. 8). Co-administration of 200 mg/kg/day metformin with 10 mg/kg/day CQ i.p. for 6 successive days before induction of ischemia and promptly at the onset of reperfusion to rats inhibited the effect of metformin on eNOS (Fig. 7), HO-1 (Fig. 9), CSE (Fig. 10), and Beclin-1 (Fig. 11) protein expressions in the liver tissue. The same combination elevated iNOS protein expression (Fig. 8) in the liver tissue.

## Discussion

Hepatocyte injury following I/R was confirmed by a considerable increase in liver enzymes: aspartate aminotransferase (AST) and alanine aminotransferase (ALT) and by histopathological alterations (Li et al. 2023).

In this study, hepatic I/RI were investigated utilizing a 70% partial hepatic warm ischemia rat model. In this model of warm hepatic I/R, liver damage was assessed both biochemically and histopathologically. Our results demonstrate that hepatic I/RI produces a significant increase in the serum AST and ALT levels.

Also, Gultekin et al. (2022) and Kartal et al. (2023) reported that in hepatic I/RI the histopathological features, vascular and sinusoid congestion, vacuolization, hepatocyte degeneration, and inflammatory infiltration were observed. In our study, the histopathological examination of liver tissue of rats subjected to I/RI showed similar marked structural damage and changes.

Because many findings proposed a link between oxidative stress (Jiang et al. 2023; Xia et al. 2024), inflammation (Li et al. 2023), apoptosis (Zaki et al. 2018; Parvizi et al. 2020), and hepatic I/RI, we investigated this hypothesis in our study. By elevating the hepatic MDA level and lowering the intracellular GSH level, this study found that the hepatic I/RI in rats was linked to oxidative stress. Additionally, rats subjected to hepatic I/RI showed elevated levels of TNF-α and caspase-3 in their liver tissue. Thus, oxidative stress, inflammation, and apoptosis may play the pivotal role in hepatic I/RI in rats.

It has been reported that treatment with metformin downregulated inflammation, apoptosis, and upregulated antioxidant enzymes in the testes (Nna et al. 2020) and in nonalcoholic fatty liver disease (Yasmin et al. 2021) in rats. In addition, Naghdi et al. (2022) hypothesized that metformin may exhibit cardioprotective effects by attenuating inflammation and oxidative stress. Furthermore, metformin produced neuroprotective and antidementia properties in male rats through attenuation of hippocampal levels of nitrite, MDA, ROS, TNF-α, and caspase-3 activity (Khaleghi-Mehr et al. 2023).

Our findings are consistent with these observations and show that treatment with metformin inhibited the development of the liver function and structure impairments in rats subjected to hepatic I/RI. In addition, metformin inhibited the development of oxidative stress, nitrosative stress, inflammation, and apoptosis in the liver tissue of these rats.

In light of the considerations that NO derived by eNOS shows a protective role (Lee et al. 2021), while excess NO generated by iNOS exacerbates hepatic I/RI (Zhang et al. 2022b) and hepatic I/R caused downregulation of eNOS and upregulation of iNOS expressions (Hassan et al. 2021; Samuvel et al. 2023), we explored NO’s potential role in hepatic I/RI. Data of our study indicate that in rats subjected to hepatic I/RI, the impairment of the hepatic function and structure was linked with an increase in the nitrite level in the liver tissues. Immunostaining of the liver tissues showed an elevation in iNOS protein expression and a reduction in eNOS protein expression. The hepatoprotective effect of metformin in I/RI in this study was linked to a reduction in NO overproduction in the liver tissue. Immunostaining of the liver tissue showed that iNOS protein expression was decreased and that of eNOS was increased after metformin treatment. Thus, NO may contribute to the development and progression of hepatic I/RI as well as metformin’s hepatoprotective effect.

To further clarify, the potential role of NO in metformin’s protective action against hepatic I/RI, the effects of NO modulators on metformin action were examined. According to our findings, the metformin’s inhibitory effect on hepatic I/RI-induced liver functional and structural impairments, oxidative stress, nitrosative stress, inflammation, and apoptosis was enhanced by concomitant administration of L-NAME, a non-selective NO synthase inhibitor (Homayoun et al. 2003). The ameliorated effect of metformin on I/RI-induced reduction of the hepatic HO-1 and H_2_S levels, inhibition eNOS, HO-1 and CSE protein expression, and a rise in iNOS protein expression was potentiated by concomitant administration of L-NAME. The metabolic precursor for NO biosynthesis, L-A (Stevens et al. 1996) produced the opposite effects of L-NAME. As a result, the metformin’s ability to protect against hepatic I/RI is due to its capability to reduce NO overproduction and hepatic iNOS protein expression.

The HO-1/carbon monoxide (CO) system has been assessed for the development of novel therapies against I/RI (Kou et al. 2020). It has been reported that treatment of rats with exogenous CO gas has beneficial effects in several disease models because of its antioxidant, anti-inflammatory, and antiapoptotic properties (Adach and Olas 2019). However, gaseous CO’s application is limited due to its harmful effect on cellular respiration. The CO-releasing molecules (CORMs) that induce low or minimal production of carboxyhemoglobin are thus regarded a safer alternative to CO gas inhalation. (Adach and Olas 2019). Furthermore, CORM-A_1_, a water-soluble CO releaser that releases CO at a substantially slower rate under physiological conditions (slow releaser), is effective in the treatment of chronic illnesses that require CO to be administered in a carefully controlled manner (Iqbal et al. 2021).

Considering these findings, the potential role of CO in hepatic I/RI was investigated in this study. According to our results, the hepatic HO-1 level and protein expression in rats subjected to hepatic I/RI were decreased. The inhibitory effect induced by I/RI on hepatic HO-1 level and protein expression was ameliorated in animals treated with metformin. Co-administration of CORM-A_1_ enhanced the inhibitory effects of metformin on I/RI-induced functional and structural impairments, oxidative stress, nitrosative stress, inflammation, and apoptosis in the liver. It improved the ameliorated effect of metformin on I/RI-induced reduction in the hepatic HO-1 and H_2_S levels, inhibition of the hepatic eNOS, HO-1 and CSE protein expression, and an increase in iNOS protein expression. On the other hand, ZnPP, which is an HO-1 inhibitor (He et al. 2011), had the opposite effects. Thus, the most likely explanation of our findings is the protective effects of metformin and metformin in combination with CORM-A_1_ in hepatic I/RI in rats which can be linked to increased HO-1 level and expression and consequently CO production. In agreement with (Kim et al. 2008), increasing CO production resulted in an increase in eNOS and CSE expressions and increase in H_2_S production while decreasing NO production and iNOS activity and expression.

Since an increasing number of studies suggest that H_2_S could protect against hepatic I/RI in several ways, such as antioxidation, anti-inflammation, and antiapoptosis (Chen et al. 2023), we investigated potential role of H_2_S in hepatic I/RI. Our findings show that I/R was associated with reduction of H_2_S level along with decrease in CSE protein expression in the liver tissue of rats. Treatment of these animals with metformin inhibited I/R-induced decline in the hepatic H_2_S level and protein expression of CSE in the rat liver tissue. Co-administration of the H_2_S doner, NaHS, enhanced the inhibitory effects of metformin on I/RI-induced functional and structural impairments, oxidative stress, nitrosative stress, inflammation, and apoptosis. NaHS enhanced the inhibitory effect of metformin on I/RI-induced reduction in the HO-1 and H_2_S levels, eNOS, HO-1, and CSE protein expressions and increase in the protein expression of iNOS. DL-PAG produced the opposite effects.

Thus, the beneficial effects of metformin in hepatic I/RI can be related to increased H_2_S levels and CSE expression in the liver tissue. H_2_S in turn inhibits excessive production of NO and the expression of iNOS while increasing the concentration and expression of eNOS, HO-1, and consequently CO levels, which suggested a crosstalk between these gasotransmitters. In agreement with many authors (Abdel-Zaher et al. 2021), inhibition of NO overproduction and increasing CO and H_2_S levels prevented oxidative stress, nitrosative stress, inflammation, and apoptosis in liver tissue of rats subjected to I/RI.

Under physiological conditions, hepatocytes have a constitutive, low degree of autophagy, which is critical for maintaining normal liver function. Under pathological situations, autophagy dysfunction is characterized by the inability to remove damaged organelles or debris (Dar et al. 2019). It has been revealed that autophagy was contributed to different liver diseases (Ueno and Komatsu 2017). Induction of autophagy can protect animals against hepatic I/RI (Liu et al. 2016). Thus, restoration or augmentation of autophagy may be a new treatment strategy to improve liver function following I/R (Rezq et al. 2021). In addition, it has been found that enhancement of autophagy-related genes (the microtubule-associated protein 1 light chain (LC3-II) and Beclin-1 (the protein markers for autophagy) expressions in liver tissue provided hepatic protection in I/RI (Zhou et al. 2021).

Wang et al. (2016) reported that metformin in spinal cord injury (SCI) enhanced the expression of Beclin-1 and LC3-II and reduced the caspase-3 level and phosphorylation levels of the mammalian target of rapamycin (mTOR) protein, which is a known inhibitor of autophagy and inhibits by rapamycin. Also, Guo et al. (2018) reported that metformin acts as a neuroprotective agent following SCI in rats by enhancing autophagy and inhibiting apoptosis. In addition, Zilinyi et al. (2018) discovered that the metformin’s cardioprotective effect in doxorubicin-induced cardiotoxicity in rats due to induction of autophagy. Similarly, metformin was found to alleviate neuropathic pain by stimulating autophagy flux (Weng et al. 2019). Moreover, Sun et al. (2021) reported that metformin may reduce diabetic nephropathy damage via regulation of the AMPK-mTOR-autophagy axis.

In our study, the hepatic I/RI decreased the Beclin-1 protein expression in the rat liver tissues. Also, the immunohistochemical analysis of the liver tissue showed that the protein expression of Beclin-1 was increased on metformin treatment. Thus, autophagy might play a role in the development and progression of hepatic I/RI and in the hepatoprotective effect of metformin.

The effects of autophagy modulators, rapamycin, which is an autophagy stimulant (Lee et al. 2016), and chloroquine, which is an autophagy inhibitor (Zhao et al. 2016), on metformin’s effect were investigated to further clarify the potential role of autophagy in its protective effect against hepatic I/RI. According to our findings the metformin’s inhibitory effect on hepatic I/RI-induced liver functional and structural impairments, oxidative stress, nitrosative stress, inflammation, and apoptosis was enhanced by concomitant administration of rapamycin. The ameliorated effect of metformin on I/RI-induced reduction of the hepatic HO-1 and H_2_S levels, inhibition of Beclin-1, eNOS, HO-1 and CSE protein expression and increase of iNOS protein expression was potentiated by concomitant administration of rapamycin. Chloroquine produced the opposite effect. As a result, the metformin’s ability to protect against hepatic I/RI can be ascribed to its ability to enhance autophagy and hepatic expression of Beclin-1.

## Conclusion

In conclusion, our results suggest that metformin inhibited I/RI-induced impairment of hepatic function and structure. Metformin produced this protective effect due to inhibition of I/RI-induced oxidative stress, nitrosative stress, inflammation, apoptosis, autophagy inhibition, NO overproduction, and decrease in CO and H_2_S levels in the liver tissue. Through inhibiting NO overproduction and raising CO and H_2_S levels and enhancement of autophagy, L-NAME, and CORM-A_1_, NaHS and RAPA potentiated the protective effect of metformin. NO donor, CO, and H_2_S biosynthesis inhibitors and autophagy inhibitor antagonized the hepatoprotective effect of metformin. Thus, there is an interrelationship between hepatic I/RI, metformin, gasotransmitters, and autophagy. In addition, gasotransmitters and autophagy appear to have a pivotal role in the development and progression of hepatic I/RI and the hepatoprotective effect of metformin against it.

## Data Availability

All source data for this work (or generated in this study) are available upon reasonable request.
